# Differential Diagnosis of Common Diseases of the Eyes

**Published:** 1894-05

**Authors:** 


Differential Diagnosis of Common Diseases of the Eyes. By W. F. Conners, M. D. Oil City, Pa. Price, postpaid, 50 cents.

This publication is a chart in which all the common diseases of the eye are arranged in a tabular form for ready reference. It is designed for the use of general physicians who have not made a special study of eye diseases, and have not therefore an expert knowledge and are not provided with instruments. It can be fastened to the wall of the office with thumb tacks or put into a frame.

